# Assessment of the Role of IQ in Associations Between Population Density and Deprivation and Nonaffective Psychosis

**DOI:** 10.1001/jamapsychiatry.2020.0103

**Published:** 2020-03-11

**Authors:** Gemma Lewis, Jennifer Dykxhoorn, Håkan Karlsson, Golam M. Khandaker, Glyn Lewis, Christina Dalman, James B. Kirkbride

**Affiliations:** 1 Division of Psychiatry, Faculty of Brain Sciences, University College London, London, United Kingdom; 2 Department of Neuroscience, Karolinska Institutet, Stockholm, Sweden; 3 Department of Psychiatry, University of Cambridge, Cambridge, United Kingdom; 4 Cambridgeshire and Peterborough NHS (National Health Service) Foundation Trust, Cambridge, United Kingdom; 5 Department of Public Health Sciences, Karolinska Institutet, Stockholm, Sweden; 6 Center for Epidemiology and Community Medicine, Stockholm County Council, Stockholm, Sweden

## Abstract

**Importance:**

Being born or raised in more densely populated or deprived areas is associated with increased risk of nonaffective psychosis in adulthood, but few studies to date have examined the role of general cognitive ability in these associations.

**Objective:**

To investigate whether lower IQ contributed to the association between population density or deprivation and nonaffective psychosis (mediation) and whether these associations were stronger in people with lower IQ (effect modification).

**Design, Setting, and Participants:**

This prospective cohort study evaluated a population-based sample of men born in Sweden from January 1, 1982, to December 31, 1988, and conscripted into military service at 18 years of age. Data were collected from January 1, 1982, to December 31, 2016, and analyzed from May 1 to December 31, 2018.

**Exposures:**

Continuous measures of small area-level population density (persons per square kilometer) and socioeconomic deprivation at birth. Deprivation was based on area-level social, criminal, and unemployment data. IQ was assessed during conscription at 18 years of age (mean [SD] IQ, 100 [15]).

**Main Outcomes and Measures:**

First diagnosis of *International Statistical Classification of Diseases and Related Health Problems, Tenth Revision*, nonaffective psychosis from 18 years of age until December 31, 2016, recorded in the National Patient Register.

**Results:**

The study sample included a total of 227 429 men who were classified as at risk of psychosis from 18 years of age until the end of follow-up. Of these, 1596 men (0.7%) were diagnosed with nonaffective psychosis. After adjustments for confounders, odds of nonaffective psychosis increased per 1-SD increase in population density (odds ratio [OR], 1.07; 95% CI, 1.04-1.14) and deprivation (OR, 1.09; 95% CI, 1.02-1.13) at birth. IQ was negatively associated with deprivation after adjustments (effect estimate per 1-SD increase in deprivation: −0.70 points; 95% CI, −0.78 to −0.62 points) but not with population density. In mediation analyses, based on the potential outcome framework, 23% (95% CI, 17%-49%) of the total effect of deprivation on nonaffective psychosis was mediated by IQ. IQ did not modify associations between deprivation or population density and nonaffective psychosis.

**Conclusions and Relevance:**

These findings suggest that being born in more deprived neighborhoods may partly increase risk of nonaffective psychosis through subsequent effects on cognitive development, consistent with the wider literature on neurodevelopmental delays associated with psychotic disorder. Identifying factors in deprived environments that give rise to this process could inform public health strategies to prevent nonaffective psychosis.

## Key Points

**Question:**

Does lower IQ contribute to the association between being born into more densely populated or deprived areas and subsequent nonaffective psychosis, and are those associations stronger in people with lower IQ?

**Findings:**

In a nationwide, population-based cohort of 227 429 Swedish men, being born in more densely populated or deprived areas was associated with an increased risk of adult nonaffective psychosis. Strong evidence suggested that being born in more deprived areas was associated with reduced IQ at 18 years of age, which could account for as much as 23% of the association between deprivation and nonaffective psychosis, but no evidence of effect modification by IQ was found.

**Meaning:**

These findings suggest that being born in more deprived neighborhoods may partly increase risk of nonaffective psychosis in adult men through its effects on cognitive development, consistent with the wider literature on neurodevelopmental delays associated with psychotic disorder.

## Teaser

This cohort study investigates whether lower IQ contributed to the association between population density or deprivation and nonaffective psychosis and whether these associations were stronger in people with lower IQ among Swedish military conscripts.

## Introduction

Studies have found that the risk of nonaffective psychosis is higher in people born, brought up, and living in more urban and deprived settings. These studies were primarily performed in Northern Europe and North America.^1,2,3,4,5,6^ Although recent cross-sectional data on psychotic experiences^7^ and disorder^8^ suggest rural-urban differences in psychosis risk may not be universal, longitudinal evidence in other contexts is missing, and there is a need to better understand the mechanisms that underpin the associations where they have been observed.^9^ Mechanisms appear to preclude individual social drift,^5^ although evidence is equivocal as to whether shared familial factors are responsible.^10,11,12,13^

Evidence from mendelian randomization studies supports a causal, bidirectional association between cognition and schizophrenia, which may share a common genetic basis.^14^ Given strong evidence that nonaffective psychoses have neurodevelopmental antecedents^15,16^ and that a lower premorbid IQ shows a dose-response relationship with subsequent risk of nonaffective psychosis,^17,18^ growing up in more urban or deprived environments could possibly lead to neurodevelopmental sequelae that affect IQ and later risk of disorder. Although IQ is heritable and stable from early adolescence to old age,^19^ strong evidence suggests that it is influenced by socioeconomic status and education in early life,^14,20,21,22,23^ which may be affected by fewer resources and educational opportunities in deprived neighborhoods.^21^ Similarly, birth and upbringing in more densely populated environments may confer greater exposure to infections,^24^ obstetric complications, or social stressors,^25^ all associated with nonaffective psychosis and all of which could influence neurocognitive development. It is therefore possible that being born into more deprived or densely populated environments might impede cognitive development, increasing subsequent risk of nonaffective psychosis. To our knowledge, no study to date has tested whether IQ lies on this pathway as a mediator of these associations.

Higher IQ might also protect against environmental risk factors for nonaffective psychosis via greater cognitive reserve or neural resilience.^17,26^ If this is true, we would expect a stronger association between urbanicity at birth and nonaffective psychosis in individuals with lower compared with higher IQ (ie, effect modification). To our knowledge, only 1 study has investigated this association, in a population-based cohort of Danish men,^27^ and found no evidence of such effect modification. However, this smaller study relied on approximate markers of urbanicity (settlement size) and lacked detailed small area-level characteristics.

We sought to test the mediating and moderating role of IQ in associations between population density and deprivation at birth and subsequent risk of nonaffective psychosis. We hypothesized that IQ would partially mediate associations between neighborhood characteristics at birth and risk of adult nonaffective psychotic disorder. We also hypothesized that IQ would modify these associations, with a stronger effect of urbanicity on psychosis risk in men with lower IQ, consistent with the cognitive reserve hypothesis.

## Methods

### Study Design and Setting

Using Psychiatry Sweden, a comprehensive record linkage for research on mental health, we identified a prospective cohort of men born in Sweden from January 1, 1982, to December 31, 1988, who were conscripted into military service at their 18th birthdays and followed up until December 31, 2016. Only 2% to 3% of the male population were excused from conscription, mostly for severe mental or physical disability. Participants were linked to national register data using a civic registration number assigned at birth. Although conscription was mandatory in Sweden from 1901 to 2010, we restricted our cohort to those born from 1982 to 1988, to coincide with availability of small area characteristics from 1982 onward, and because conscription began to decline for birth cohorts after 1988, leading to less representative samples. Men diagnosed with nonaffective psychosis before conscription were excluded. This study was approved by the ethical review board at Karolinska Institutet, Stockholm, Sweden, which waived the need for informed consent for use of registry data. This study followed the Strengthening the Reporting of Observational Studies in Epidemiology (STROBE) reporting guideline.

### Outcome

Our outcome was first diagnosis of an *International Statistical Classification of Diseases and Related Health Problems, Tenth Revision,* nonaffective psychosis (codes F20-F29) recorded in the National Patient Registry from 18 years of age (earliest, January 1, 2000) until December 31, 2016. The National Patient Register had 100% coverage of inpatient care in Sweden during this period, with outpatient coverage beginning in 2001 and complete by 2006.^28^

### Exposures

Our main exposures were population density and socioeconomic deprivation at birth, at the Small Area Marketing Statistics (SAMS) area level (9200 SAMS regions; median 2011 population size, 726 [interquartile range, 312-1378]),^29^ identified via the Statistics Sweden Regional Register, which contains annually updated information on neighborhood characteristics since 1982. SAMS regions are designed to maximize internal homogeneity with respect to socioeconomic and demographic characteristics. Place of birth was the mother’s residence when her child was born.

We estimated population density as people per square kilometer in each participant’s SAMS region in their birth year (eFigure 1 in the Supplement). We estimated deprivation for each participant’s SAMS region in their birth year based on a composite measure of levels of crime and unemployment, low income, and receipt of social benefits (eMethods 1 in the Supplement). We divided both exposures by their SDs to align them on comparable scales.

### Mediators and Moderators

Data on IQ was obtained from the military conscription register at approximately 18 years of age (mean [SD] age, 18.22 [0.41] years) (details in eMethods 2 in the Supplement). IQ scores were standardized and categorized to give a normally distributed variable ranging from 1 to 9, which we transformed to have a mean of 100 and SD of 15.^24,30^ We divided this scale by the SD.

### Confounders

We included potential confounders from linked registers (eMethods 1 in the Supplement). These confounders consisted of paternal age at birth, parental history of severe mental illness (yes/no), family disposable income quintile at participant birth, parental educational level, migrant status, and paternal IQ (obtained from the military conscription register).

### Missing Data

Based on previous register studies, we expected little missing data (<5%), although conscription IQ data has more missing values (17%) (eFigure 2 in the Supplement).^24^ We handled missing data via multiple imputation in sensitivity analyses (eMethods 3 in the Supplement). To impute missing data, we used conscripts’ educational attainment at 16 years of age, all characteristics described in Tables 1 and 2, and several auxiliary variables (maternal smoking, obstetric complications and infections in the first year of the participants’ life, and childhood residential mobility) (eTables 8 and 9 in the Supplement).

**Table 1.** Baseline Characteristics of the Sample Used for Analyses According to Categories of Population Density^a^

Characteristic	Population Density Category^b^			
	Most Rural (n = 73 558)	Semirural (n = 70 446)	Semiurban (n = 48 761)	Most Urban (n = 34 664)
Nonaffective psychosis, No. (%)	385 (0.5)	436 (0.6)	440 (0.9)	335 (1.0)
IQ score, mean (SD)	99.25 (15.18)	100.31 (15.22)	100.56 (15.34)	99.92 (15.54)
Most deprived category, No. (%)	3033 (4.1)	2743 (3.9)	7001 (14.4)	9363 (27.0)
Maternal educational level ≤9 y, No. (%)	2051 (2.8)	1726 (2.5)	1444 (3.0)	1684 (4.9)
Paternal educational level ≤9 y, No. (%)	6906 (9.4)	4156 (5.9)	2318 (4.8)	2047 (5.9)
Lowest family income quintile, No. (%)	1978 (2.7)	1297 (1.8)	1313 (2.7)	1312 (3.8)
History of any psychosis in either parent, No. (%)	1713 (2.3)	1782 (2.5)	1578 (3.2)	1322 (3.8)
Parent(s) migrated into Sweden, No. (%)	5441 (7.4)	7983 (11.3)	8725 (17.9)	9189 (26.5)
Mother age at birth, mean (SD), y	28.44 (4.93)	28.73 (5.01)	28.45 (5.09)	28.02 (5.04)
Father age at birth, mean (SD), y	31.51 (5.58)	31.44 (5.64)	31.10 (5.84)	30.84 (6.00)

^a^ Includes participants with no missing data (n = 227 429).

^b^ Units are number of people per square kilometer. Categories were created using the population density distribution in the entire Swedish population: 0 to 152.9 (minimum to median value; most rural); 153.0 to 1488.7 (median to 75th centile; semirural); 1488.8 to 4590.1 (75th to 90th centiles; semiurban); and 4590.2 to 48 657.0 (90th centile to the maximum value; most urban). The *P* values for categorical variables are calculated from χ^2^ tests; for continuous variables, the global *P* values are calculated from linear regressions with population density the exposure. *P* < .001 for all comparisons.

**Table 2.** Baseline Characteristics of the Sample Used for Analyses According to Categories of Socioeconomic Deprivation^a^

Characteristic	Socioeconomic Deprivation Category^b^			
	Low (n = 113 659)	Low to Medium (n = 57 293)	Medium to High (n = 34 137)	High (n = 22 340)
Nonaffective psychosis, No. (%)	715 (0.6)	373 (0.7)	266 (0.8)	242 (1.1)
IQ score, mean (SD)	101.35 (15.10)	99.77 (15.21)	98.30 (15.40)	95.92 (15.36)
Most urban population density category, No. (%)	10 008 (8.8)	9038 (15.8)	6255 (18.3)	9363 (41.9)
Maternal educational level ≤9 y, No. (%)	2297 (2.0)	1581 (2.8)	1155 (3.4)	1872 (8.4)
Paternal educational level ≤9 y, No. (%)	6369 (5.6)	4261 (7.4)	2616 (7.7)	2181 (9.8)
Lowest family income quintile, No. (%)	2215 (1.9)	1591 (2.8)	1064 (3.1)	1030 (4.6)
History of any psychosis in either parent, No. (%)	2708 (2.4)	1575 (2.7)	1119 (3.3)	993 (4.4)
Parent(s) migrated into Sweden, No. (%)	12 318 (10.8)	6463 (11.3)	5117 (15.0)	7440 (33.3)
Mother age at birth, mean (SD), y	29.22 (4.85)	28.24 (4.95)	27.58 (5.06)	26.62 (5.13)
Father age at birth, mean (SD), y	31.89 (5.52)	31.14 (5.70)	31.58 (5.89)	29.75 (6.11)

^a^ Includes participants with no missing data (n = 227 429).

^b^ Units are based on the deprivation index. Categories were created using the deprivation scores in the entire Swedish population: −4.2 to −0.3 (minimum to median; low); −0.4 to 0.9 (median to 75th centile; low to medium); 1.0 to 2.5 (75th to 90th centile; medium to high); and 2.6 to 34.2 (90th centile to maximum; high). The *P* values for categorical variables are calculated from χ^2^ tests; for continuous variables, the global *P* values are calculated from linear regressions with deprivation the exposure. *P* < .001 for all comparisons.

### Statistical Analysis

Data were analyzed from May 1 to December 31, 2018. Full details of the statistical methods are given in the eMethods 1 in the Supplement. Briefly, we used multilevel logistic regression to examine associations between population density and deprivation and the odds of nonaffective psychosis. We tested for effect modification by IQ in these models by fitting interaction terms between each exposure and IQ, assessed via likelihood ratio test. Power analysis via simulation was conducted for effect modification between population density at birth and IQ and between deprivation at birth and IQ, simultaneously on risk of nonaffective psychosis (eMethods 4 and eTable 12 in the Supplement). We also calculated the population attributable fraction for population density and deprivation on nonaffective psychosis. Where appropriate, we tested for mediation by IQ of the association between population density or deprivation and nonaffective psychosis risk using the potential outcomes framework, a class of causal mediation analysis fitted using parametric mediation models (eMethods 2 in the Supplement).^31,32^ We performed sensitivity analyses to investigate whether any observed associations were explained by prodromal effects of psychosis on IQ, paternal IQ, and missing data. All analyses were performed in Stata, version 15 (StataCorp LLC).

## Results

Our complete case sample included 227 429 conscripted men born in Sweden from 1982 to 1988 (80.2% of eligible sample) (eFigure 2 in the Supplement). Conscripts with missing data (18.8%) were more likely to come from more densely populated and deprived areas and to have a diagnosis of nonaffective psychosis, lower IQ, parents from the lowest educational and income categories, parents with a history of psychosis, and parents who had migrated to Sweden (eTable 1 in the Supplement).

In our sample, 1596 men (0.7%) were diagnosed with nonaffective psychosis. A higher proportion of participants with nonaffective psychosis were born in the most densely populated (335 of 34 664 [1.0%] vs 385 of 73 558 [0.5%]) (Table 1) and deprived (242 of 22 340 [1.1%] vs 715 of 113 659 [0.6%]) SAMS regions (Table 2) than the population at risk. IQ scores at 18 years of age were lower in the most deprived areas (Table 2), but no clear association was apparent with population density (Table 1). We found a small correlation between population density and deprivation (*r* = 0.28).

Population density and deprivation were associated with risk of nonaffective psychosis (Table 3), including in multivariable-adjusted models (odds ratio [OR] for population density, 1.07 [95% CI, 1.04-1.14]; OR for deprivation, 1.09 [95% CI, 1.02-1.13]). We found no evidence of nonlinearity for population density (*P* = .26) or deprivation (*P* = .18). We estimated a population attributable fraction of 31% (95% CI, 11%-47%) for deprivation and 6% (95% CI, 3%-9%) for population density.

**Table 3.** Univariable and Multivariable Associations Between Population Density, Deprivation, or IQ and Nonaffective Psychosis

Exposure	Nonaffective Psychosis (n = 227 429)							
	Unadjusted^a^		Bivariable^b^		Multivariable^c^		Fully Adjusted^d^	
	OR (95% CI)	*P* Value	OR (95% CI)	*P* Value	OR (95% CI)	*P* Value	OR (95% CI)	*P* Value
Population density^e^	1.17 (1.12-1.21)	<.001	1.13 (1.09-1.18)	<.001	1.07 (1.04-1.14)	.008	1.10 (1.05-1.15)	<.001
Deprivation^f^	1.18 (1.12-1.23)	<.001	1.13 (1.08-1.19)	<.001	1.09 (1.02-1.13)	.002	1.05 (1.00-1.11)	.04
IQ^g^	0.71 (0.68-0.75)	<.001	NA	NA	NA	NA	0.70 (0.67-0.74)	<.001

Abbreviations: NA, not applicable; OR, odds ratio.

^a^ Indicates separate univariable models for population density, deprivation, and IQ.

^b^ Indicates model for population density and deprivation.

^c^ Population density and deprivation are adjusted for paternal age at birth, family income, maternal educational level, paternal educational level, any psychosis or bipolar disorder in parents, and parent(s) born outside Sweden.

^d^ Adjusted for the variables in the above multivariable model and IQ. This model was then run again with interaction terms for population density and IQ and for deprivation and IQ.

^e^ Units are number of people per square kilometer, and unit change is per 1 SD (3863.97 people per square kilometer).

^f^ Units are based on the deprivation index, and unit change is per 1 SD (1.96 points).

^g^ Units are based on the Wechsler Adult Intelligence Scale score, and unit change is per 1 SD (15 points).

We found strong evidence of a negative association between IQ and nonaffective psychosis, which remained after adjustments (OR for 1-SD increase in IQ, 0.70; 95% CI, 0.67-0.74) (Table 3). There was no evidence that the association between IQ and nonaffective psychosis was nonlinear (*P* = .57). There was no evidence that IQ modified associations between population density (likelihood ratio test, *P* = .52) or deprivation (likelihood ratio test, *P* = .65) and nonaffective psychosis.

In a univariable model, IQ scores increased by 0.15 (95% CI, 0.03-0.26) points per 1-SD increase in population density, but this association disappeared after adjustments (0.06 [95% CI, −0.02 to 0.14] points per 1-SD increase) (Table 4). IQ scores changed by −0.70 (95% CI, −0.78 to −0.62) points per 1-SD increase in deprivation in the fully adjusted model (Table 4).

**Table 4.** Univariable and Multivariable Change in IQ Score According to Population Density and Deprivation^a^

Exposure Variable	IQ Score (n = 227 429)					
	Unadjusted^b^		Bivariable^c^		Fully Adjusted^d^	
	Change in IQ Score (95% CI)	*P* Value	Change in IQ Score (95% CI)	*P* Value	Change in IQ Score (95% CI)	*P* Value
Population density^e^	0.15 (0.03 to 0.26)	.01	0.61 (0.50 to 0.72)	<.001	0.06 (−0.02 to 0.14)	.18
Deprivation^f^	−1.58 (−1.68 to −1.48)	<.001	−1.72 (−1.82 to −1.62)	<.001	−0.70 (−0.78 to −0.62)	<.001

^a^ See path A of mediation model, eFigure 3 in the Supplement.

^b^ Indicates separate univariable models for population density and deprivation.

^c^ Indicates bivariable model for population density and deprivation.

^d^ Population density and deprivation are adjusted for paternal age at birth, family income, maternal educational level, paternal educational level, any psychosis or bipolar disorder in parents, and parent(s) born outside Sweden.

^e^ Units are number of people per square kilometer, and unit change is per 1 SD (3863.97 people per square kilometer).

^f^ Units are based on the deprivation index, and unit change is per 1 SD (1.96 points).

Given the absence of evidence for an association between population density and IQ (path A in eFigure 3 in the Supplement and Table 4), we restricted mediation analysis to deprivation. After adjustments, the OR for the total effect of deprivation on nonaffective psychosis was 1.08 (95% CI, 1.03-1.13), constituting a natural indirect effect of 1.02 (95% CI, 1.01-1.02) and a natural direct effect of 1.06 (95% CI, 1.01-1.11) (Table 5). This was equivalent to 23% (95% CI, 17%-49%) of the total effect of deprivation on nonaffective psychosis being attributable to changes in IQ, if associations were causal.

**Table 5.** Univariable and Multivariable Mediation Analyses of the Association Between Population Density/Deprivation at Birth and Risk of Nonaffective Psychosis by IQ at 18 Years of Age^a^

Pathways and Mediation Effects	Unadjusted Model		Fully Adjusted Model^b^	
	Effect Estimate (95% CI)	*P* Value	Effect Estimate (95% CI)	*P* Value
Deprivation				
Path A (exposure-mediator)^c^	−1.58 (−1.68 to −1.48)	<.001	−0.70 (−0.78 to −0.62)	<.001
Path B (mediator-outcome)^d^	0.71 (0.68 to 0.75)	<.001	0.70 (0.67 to 0.74)	<.001
Path C (exposure-outcome)^e^	1.18 (1.12 to 1.23)	<.001	1.07 (1.02 to 1.13)	.005
Mediation model				
Total effect	1.19 (1.14 to 1.23)	<.001	1.08 (1.03 to 1.13)	.001
Natural direct effect	1.14 (1.10 to 1.19)	<.001	1.06 (1.01 to 1.11)	.01
Natural indirect effect	1.04 (1.03 to 1.04)	<.001	1.02 (1.01 to 1.02)	<.001
Mediation by IQ, %	22 (21 to 25)	NA	23 (17 to 49)	NA

Abbreviation: NA, not applicable.

^a^ Includes participants with no missing data (n = 227 429).

^b^ Adjusted for paternal age at birth, family income, maternal educational level, paternal educational level, any psychosis or bipolar disorder in parents, and parent(s) born outside of Sweden.

^c^ Indicates change in IQ Score at 18 years of age, per SD increase in deprivation at birth.

^d^ Indicates odds of nonaffective psychosis, per SD increase in IQ at 18 years of age.

^e^ Indicates odds of nonaffective psychosis, per SD increase in deprivation at birth.

In sensitivity analyses excluding those diagnosed within 2 years of conscription (n = 142) (eTables 2 and 10 in the Supplement) or additionally adjusting for paternal IQ (144 576 [64.0% of analytic sample]) (eTable 3 in the Supplement), effect estimates for associations of population density, deprivation, and IQ with nonaffective psychosis were unaltered (eTable 2 in the Supplement). The association between deprivation and IQ reduced from −0.70 (95% CI, −0.78 to −0.62; *P* < .001) to −0.28 (95% CI, −0.36 to −0.20; *P* < .001) after adjustment for paternal IQ (path A, eFigure 3 and eTable 4 in the Supplement). In the multiply imputed sample, this association after adjusting for paternal IQ was −0.44 (95% CI, −0.52 to −0.36; *P* < .001) (eTable 5 in the Supplement). In mediation analyses adjusted for paternal IQ, estimates were 1.08 (95% CI, 1.01-1.15) for total effects, 1.01 (95% CI, 1.01-1.01) for indirect effects, and 1.07 (95% CI, 1.01-1.15) for direct effects. This equated to 9% (95% CI, 7%-35%) mediation.

Multiple imputation analyses used a sample of 282 232 (99.5% of those eligible) (eFigure 2 in the Supplement), excluding 1357 men missing SAMS at birth, required for multilevel models. Multiply imputed estimates were unaltered (eTables 6 and 7 in the Supplement). Multiply imputed analyses adjusted for paternal IQ were also similar (eTable 11 in the Supplement).

## Discussion

### Main Findings

In this large, prospective cohort of Swedish men, we demonstrate for the first time, to our knowledge, that as much as 23% of the risk of nonaffective psychosis associated with deprivation at birth could be attributable to indirect effects on cognition, measured by IQ at 18 years of age, if these associations are causal. We found no evidence that population density at birth was associated with IQ, and therefore no evidence of mediation in this pathway. Population density, as with deprivation and consistent with earlier studies,^1,2,3,4,5,6^ was independently associated with risk of nonaffective psychosis in adulthood. We found no evidence that IQ modified these associations.

### Comparison With Previous Studies

Our finding that deprivation at birth was associated with lower IQ later in development is consistent with a previous UK twin study.^21^ That study found a larger effect size, reasons for which are unclear, although differences in deprivation measurement and context may contribute. Inequalities in schooling and other social factors may be smaller in Sweden than in the United Kingdom. If our results generalize to more deprived contexts, the proportion of nonaffective psychosis mediated by IQ may be greater. The absence of effect modification between population density at birth or deprivation and IQ on nonaffective psychosis suggests that exposure to worse social environments in early life has a similar association with nonaffective psychosis risk for all people independent of IQ, and from this perspective our results do not support theories of cognitive reserve or neural resilience.^17,26^ These theories suggest that people with higher IQ respond better to social adversity compared with people of lower IQ.^17,26^

### Potential Mechanisms

Being born and raised in a more deprived area might contribute to lower IQ in several ways. Children raised in more deprived areas could experience fewer opportunities and resources for cognitive engagement and learning,^21^ affecting the quality and duration of education. Strong evidence suggests that educational level is causally related to higher IQ. Quality of education might also influence IQ, although more research is needed.^33^ Deprivation could also affect cognitive development via increased exposure to obstetric complications, poorer nutrition, infections in early life,^24^ or increased exposure to substance misuse (eg, cannabis); these factors have been associated with psychosis risk.^24,34^ Future studies should investigate whether these factors also mediate causal pathways between deprivation and nonaffective psychosis. Future studies could also disaggregate the composite deprivation measure to investigate which components are most strongly associated nonaffective psychosis.

The association between population density and psychosis was independent of deprivation and IQ, suggesting that these factors do not cause urban-rural differences in psychosis incidence. Given the presence of unmediated direct effects between population density/deprivation at birth and subsequent risk of nonaffective psychosis in our data, we believe that other mechanisms must operate. Our finding that IQ does not mediate associations between population density and nonaffective psychosis suggests that other mechanisms must be implicated. Social stress paradigms may be relevant,^35^ potentially via disruption to neurobiological pathways, including the dopaminergic system, as apparent in animal and human studies.^16^ If birth and upbringing in more deprived or densely populated environments expose people to more stimuli requiring threat monitoring, this may result in paranoid attributional styles or other psychotic phenomena.^36,37^

### Strengths and Limitations

Our study used a large prospective cohort, linked to reliable register data on small area characteristics at birth and IQ, along with valid diagnoses of nonaffective psychosis. We included all cases from inpatient and outpatient settings in Sweden from 2000 through 2016. Studies using inpatient data alone may have substantial underascertainment.^38^ Although we might have missed some men presenting as outpatients from 2001 through 2005 when coverage was incomplete,^28^ we do not believe that this will have appreciably altered our interpretations.

Sensitivity analyses using multiple imputation for missing data did not alter our findings. Given this, it seems unlikely that results from mediation analyses, for which we could not run multiple imputation, would have been altered. We used multilevel models to investigate main effects and effect modification, but these are also not yet widely available for causal mediation analysis.^39^ Our sample was broadly representative of men born in Sweden during our inclusion period, because few men were exempt from conscription at this time.^40^ Our findings may not generalize to women, although previous studies find no evidence that associations of population density^5,6^ or IQ^17^ with nonaffective psychosis vary by sex. Excluding individuals who developed nonaffective psychosis before 19 years of age may also have affected generalizability.

Our use of the potential outcomes approach for mediation makes the strong assumption^41^ that those with and without exposure or mediators are substitutable, conditioned on confounders. We adjusted for several potential familial, parental, and demographic confounders. Nonetheless, we cannot exclude the possibility that genetic or environmental factors influenced selection into more densely populated or deprived areas.^10,13^ If these factors also influenced cognition and later risk of nonaffective psychosis, this might have led to unobserved confounding.^13,42,43^

We were unable to adjust for common mental health problems before conscription, such as depression and anxiety, which might have confounded associations between IQ and nonaffective psychosis. We excluded people with psychosis before conscription. Our sensitivity analysis excluding participants diagnosed with nonaffective psychosis within 2 years after conscription would also partially control for this. Furthermore, studies have identified cognitive development in childhood, when depression and anxiety are rare and unlikely to confound, as a risk factor for later nonaffective psychosis.^6^ Data on cannabis use are also unavailable in the Swedish registries. Cannabis use might have confounded the association between IQ and nonaffective psychosis.

We were unable to adjust for maternal IQ (women were not conscripted during the relevant time period). We will have partially controlled for maternal IQ by adjusting for maternal educational level, given the strong association between paternal IQ and paternal educational level. We would also expect maternal IQ to be correlated with paternal IQ, which we controlled for in our analyses.

Some evidence suggested that paternal IQ partially confounded the association between deprivation and participant IQ, although the association remained present and statistically strong. Including paternal IQ also led to a substantially smaller sample (given missing data), making it difficult to differentiate among confounding, bias, and lower power. However, the confounding effect of paternal IQ highlights the importance of adjusting for confounders of the exposure and mediator in mediation analyses. There was no evidence that paternal IQ confounded the association between deprivation and nonaffective psychosis. Because adjustment for paternal IQ did not affect the exposure-outcome association, this implies that selection via this mechanism is unlikely. However, we interpret this cautiously, acknowledging other potential selection factors that may not travel via paternal IQ.

Evidence of familial confounding is equivocal. Current polygenic risk scores for schizophrenia appear to be associated with living in more densely populated environments in adulthood^8^ and perhaps adolescence^9^ but not at birth.^5,9^ By contrast, they have been consistently associated with deprivation at birth^5^ and in adulthood^8^ in the limited studies on this issue. Such genetic data are not available in Swedish registries for psychotic disorders, and polygenic risk scores capture a small amount of genetic variance; our adjustment for parental severe mental illness, and in sensitivity analyses, paternal IQ, is a strength.

While we acknowledge that odds ratios were small, the population level effect is potentially large. We estimated a population attributable fraction of 31% for deprivation. This is a considerable proportion and represents an important finding when the complexity of effect sizes is appropriately considered. In addition, we did not consider possible time-varying effects of the exposures on later psychosis risk, choosing to measure these exposures at birth, before prodromal effects could have biased our results.

## Conclusions

If replicated, our discoveries have the potential to elucidate causal pathways (neurodevelopmental, social, and biological) through which deprivation affects different cognitive domains involved in the onset of psychotic disorder. This research could provide new targets for intervention, particularly in deprived communities where strategies to promote cognitive development in children and young people could reduce the disproportionate burden of nonaffective psychoses.

## Notes

Supplement. eFigure 1. Distribution of Population Density Scores at Birth in the Sample With Complete Data Used for Analyses (n = 227 429) eFigure 2. Flow of Participants Through the Study eFigure 3. Mediation Model for the Association Between Population Density/Deprivation and Nonaffective Psychosis eMethods 1. Measures and Statistical Analysis eMethods 2. Mediation eMethods 3. Auxiliary Variables Included in Multiple Imputation Models eMethods 4. Power Simulation of Effect Modification eTable 1. Baseline Characteristics of Samples With Complete and Missing Data eTable 2. Univariable and Multivariable Odds Ratios (OR) for Associations Among Population Density, Deprivation, IQ, and Nonaffective Psychosis, Excluding Those Diagnosed Within 2 Years of Conscription (n = 142) eTable 3. Univariable and Multivariable Odds Ratios (OR) for Associations Between Population Density, Deprivation, IQ, and Nonaffective Psychosis, Further Adjusted for Paternal IQ eTable 4. Univariable and Multivariable Change in IQ According to Population Density and Deprivation Further Adjusted for Paternal IQ eTable 5. Univariable and Multivariable Change in IQ According to Population Density and Deprivation Multiply Imputed Sample and Further Adjusted for Paternal IQ eTable 6. Univariable and Multivariable Odds Ratios (OR) for Associations Between Population Density, Deprivation, IQ, and Nonaffective Psychosis in the Sample With Missing Data Replaced by Multiple Imputation eTable 7. Univariable and Multivariable Change in IQ Score According to Population Density and Deprivation in the Sample With Missing Data Replaced by Multiple Imputation eTable 8. List of Diagnostic Codes for Obstetric Complications and Early Life (0-12 Months) Infections as Recorded in the Maternal Birth Register eTable 9. List of Diagnostic Codes for Early Life (0-12 Months) Infections as Recorded in the National Patient Register eTable 10. Univariable and Multivariable Change in IQ Score According to Population Density and Deprivation, Excluding Those Diagnosed With Nonaffective Psychosis Within 2 years of Conscription (n = 142) eTable 11. Univariable and Multivariable Odds Ratios (OR) for Associations Between Population Density, Deprivation, IQ, and Nonaffective Psychosis, Multiply Imputed Sample and Further Adjusted for Paternal IQ eTable 12. Power Simulations for Interaction Between Population Density and Deprivation at Birth and IQ on Risk of Nonaffective Psychosis for Various Hypothesized Effect Sizes eReferences.

## References

[1] Vassos E, Pedersen CB, Murray RM, Collier DA, Lewis CM Meta-analysis of the association of urbanicity with schizophrenia. Schizophr Bull. 2012;38(6):1118-1123. doi:10.1093/schbul/sbs096 23015685 PMC3494055

[2] Heinz A, Deserno L, Reininghaus U Urbanicity, social adversity and psychosis. World Psychiatry. 2013;12(3):187-197. doi:10.1002/wps.20056 24096775 PMC3799240

[3] Zammit S, Lewis G, Rasbash J, Dalman C, Gustafsson J-E, Allebeck P Individuals, schools, and neighborhood: a multilevel longitudinal study of variation in incidence of psychotic disorders. Arch Gen Psychiatry. 2010;67(9):914-922. doi:10.1001/archgenpsychiatry.2010.101 20819985

[4] Kirkbride JB, Jones PB, Ullrich S, Coid JW Social deprivation, inequality, and the neighborhood-level incidence of psychotic syndromes in East London. Schizophr Bull. 2014;40(1):169-180. doi:10.1093/schbul/sbs151 23236081 PMC3885290

[5] Mortensen PB, Pedersen CB, Westergaard T, Effects of family history and place and season of birth on the risk of schizophrenia. N Engl J Med. 1999;340(8):603-608. doi:10.1056/NEJM199902253400803 10029644

[6] Sundquist K, Frank G, Sundquist J Urbanisation and incidence of psychosis and depression: follow-up study of 4.4 million women and men in Sweden. Br J Psychiatry. 2004;184(4):293-298. doi:10.1192/bjp.184.4.293 15056572

[7] DeVylder JE, Kelleher I, Lalane M, Oh H, Link BG, Koyanagi A Association of urbanicity with psychosis in low- and middle-income countries. JAMA Psychiatry. 2018;75(7):678-686. doi:10.1001/jamapsychiatry.2018.0577 29799917 PMC6145671

[8] Jongsma HE, Gayer-Anderson C, Lasalvia A, ; European Network of National Schizophrenia Networks Studying Gene-Environment Interactions Work Package 2 (EU-GEI WP2) Group Treated incidence of psychotic disorders in the multinational EU-GEI study. JAMA Psychiatry. 2018;75(1):36-46. doi:10.1001/jamapsychiatry.2017.3554 29214289 PMC5833538

[9] Kirkbride JB, Keyes KM, Susser E City living and psychotic disorders: implications of global heterogeneity for theory development. JAMA Psychiatry. 2018;75(12):1211-1212. doi:10.1001/jamapsychiatry.2018.2640 30304485

[10] Colodro-Conde L, Couvy-Duchesne B, Whitfield JB, Association between population density and genetic risk for schizophrenia. JAMA Psychiatry. 2018;75(9):901-910. doi:10.1001/jamapsychiatry.2018.1581 29936532 PMC6142911

[11] Paksarian D, Trabjerg BB, Merikangas KR, The role of genetic liability in the association of urbanicity at birth and during upbringing with schizophrenia in Denmark. Psychol Med. 2018;48(2):305-314. doi:10.1017/S0033291717001696 28659227 PMC6361630

[12] Solmi F, Lewis G, Zammit S, Kirkbride JB Neighbourhood characteristics at birth and positive and negative psychotic symptoms in adolescence: findings from the ALSPAC birth cohort [published online June 5, 2019]. Schizophr Bull. 31167032 10.1093/schbul/sbz049 PMC7147568

[13] Sariaslan A, Larsson H, D’Onofrio B, Långström N, Fazel S, Lichtenstein P Does population density and neighborhood deprivation predict schizophrenia? a nationwide Swedish family-based study of 2.4 million individuals. Schizophr Bull. 2015;41(2):494-502. doi:10.1093/schbul/sbu105 25053652 PMC4332947

[14] Savage JE, Jansen PR, Stringer S, Genome-wide association meta-analysis in 269 867 individuals identifies new genetic and functional links to intelligence. Nat Genet. 2018;50(7):912-919. doi:10.1038/s41588-018-0152-6 29942086 PMC6411041

[15] Jones P, Rodgers B, Murray R, Marmot M Child development risk factors for adult schizophrenia in the British 1946 birth cohort. Lancet. 1994;344(8934):1398-1402. doi:10.1016/S0140-6736(94)90569-X 7968076

[16] Howes OD, Murray RM Schizophrenia: an integrated sociodevelopmental-cognitive model. Lancet. 2014;383(9929):1677-1687. doi:10.1016/S0140-6736(13)62036-X 24315522 PMC4127444

[17] Khandaker GM, Barnett JH, White IR, Jones PB A quantitative meta-analysis of population-based studies of premorbid intelligence and schizophrenia. Schizophr Res. 2011;132(2-3):220-227. doi:10.1016/j.schres.2011.06.017 21764562 PMC3485562

[18] Zammit S, Allebeck P, David AS, A longitudinal study of premorbid IQ score and risk of developing schizophrenia, bipolar disorder, severe depression, and other nonaffective psychoses. Arch Gen Psychiatry. 2004;61(4):354-360. doi:10.1001/archpsyc.61.4.354 15066893

[19] Gow AJ, Johnson W, Pattie A, Stability and change in intelligence from age 11 to ages 70, 79, and 87: the Lothian birth cohorts of 1921 and 1936. Psychol Aging. 2011;26(1):232-240. doi:10.1037/a0021072 20973608

[20] Plomin R, von Stumm S The new genetics of intelligence. Nat Rev Genet. 2018;19(3):148-159. doi:10.1038/nrg.2017.104 29335645 PMC5985927

[21] von Stumm S, Plomin R Socioeconomic status and the growth of intelligence from infancy through adolescence. Intelligence. 2015;48:30-36. doi:10.1016/j.intell.2014.10.002 26640306 PMC4641149

[22] Tucker-Drob EM, Briley DA Continuity of genetic and environmental influences on cognition across the life span: a meta-analysis of longitudinal twin and adoption studies. Psychol Bull. 2014;140(4):949-979. doi:10.1037/a0035893 24611582 PMC4069230

[23] Brinch CN, Galloway TA Schooling in adolescence raises IQ scores. Proc Natl Acad Sci U S A. 2012;109(2):425-430. doi:10.1073/pnas.1106077109 22203952 PMC3258640

[24] Khandaker GM, Dalman C, Kappelmann N, Association of childhood infection with IQ and adult nonaffective psychosis in Swedish men: a population-based longitudinal cohort and co-relative study. JAMA Psychiatry. 2018;75(4):356-362. doi:10.1001/jamapsychiatry.2017.4491 29450471 PMC5875340

[25] Kelly BD, O’Callaghan E, Waddington JL, Schizophrenia and the city: a review of literature and prospective study of psychosis and urbanicity in Ireland. Schizophr Res. 2010;116(1):75-89. doi:10.1016/j.schres.2009.10.015 19897342

[26] Stern Y What is cognitive reserve? theory and research application of the reserve concept. J Int Neuropsychol Soc. 2002;8(3):448-460. doi:10.1017/S1355617702813248 11939702

[27] Toulopoulou T, Picchioni M, Mortensen PB, Petersen L IQ, the urban environment, and their impact on future schizophrenia risk in men. Schizophr Bull. 2017;43(5):1056-1063. doi:10.1093/schbul/sbw147 28338769 PMC5581890

[28] Ludvigsson JF, Andersson E, Ekbom A, External review and validation of the Swedish National Inpatient Register. BMC Public Health. 2011;11:450. doi:10.1186/1471-2458-11-450 21658213 PMC3142234

[29] Price C, Dalman C, Zammit S, Kirkbride JB Association of residential mobility over the life course with nonaffective psychosis in 1.4 million young people in Sweden. JAMA Psychiatry. 2018;75(11):1128-1136. doi:10.1001/jamapsychiatry.2018.2233 30140915 PMC6248097

[30] Hayes JF, Osborn DPJ, Lewis G, Dalman C, Lundin A Association of late adolescent personality with risk for subsequent serious mental illness among men in a Swedish nationwide cohort study. JAMA Psychiatry. 2017;74(7):703-711. doi:10.1001/jamapsychiatry.2017.0583 28538982 PMC5710245

[31] Hernán MA A definition of causal effect for epidemiological research. J Epidemiol Community Health. 2004;58(4):265-271. doi:10.1136/jech.2002.006361 15026432 PMC1732737

[32] Maldonado G, Greenland S Estimating causal effects. Int J Epidemiol. 2002;31(2):422-429. doi:10.1093/ije/31.2.422 11980807

[33] Ritchie SJ, Tucker-Drob EM How much does education improve intelligence? a meta-analysis. Psychol Sci. 2018;29(8):1358-1369. doi:10.1177/0956797618774253 29911926 PMC6088505

[34] Brown AS The environment and susceptibility to schizophrenia. Prog Neurobiol. 2011;93(1):23-58. doi:10.1016/j.pneurobio.2010.09.003 20955757 PMC3521525

[35] Mizrahi R Social stress and psychosis risk: common neurochemical substrates? Neuropsychopharmacology. 2016;41(3):666-674. doi:10.1038/npp.2015.274 26346639 PMC4707841

[36] Bentall RP, Kinderman P, Kaney S The self, attributional processes and abnormal beliefs: towards a model of persecutory delusions. Behav Res Ther. 1994;32(3):331-341. doi:10.1016/0005-7967(94)90131-7 8192633

[37] Roiser JP, Howes OD, Chaddock CA, Joyce EM, McGuire P Neural and behavioral correlates of aberrant salience in individuals at risk for psychosis. Schizophr Bull. 2013;39(6):1328-1336. doi:10.1093/schbul/sbs147 23236077 PMC3796080

[38] Jörgensen L, Ahlbom A, Allebeck P, Dalman C The Stockholm Non-affective Psychoses Study (SNAPS): the importance of including out-patient data in incidence studies. Acta Psychiatr Scand. 2010;121(5):389-392. doi:10.1111/j.1600-0447.2009.01500.x 19878139

[39] Arah OA Commentary: tobacco smoking and asthma: multigenerational effects, epigenetics and multilevel causal mediation analysis. Int J Epidemiol. 2018;47(4):1117-1119. doi:10.1093/ije/dyy193 30184137 PMC6124611

[40] David AS, Malmberg A, Brandt L, Allebeck P, Lewis G IQ and risk for schizophrenia: a population-based cohort study. Psychol Med. 1997;27(6):1311-1323. doi:10.1017/S0033291797005680 9403903

[41] Imai K, Keele L, Tingley D A general approach to causal mediation analysis. Psychol Methods. 2010;15(4):309-334. doi:10.1037/a0020761 20954780

[42] Pedersen CB, Mortensen PB Are the cause(s) responsible for urban-rural differences in schizophrenia risk rooted in families or in individuals? Am J Epidemiol. 2006;163(11):971-978. doi:10.1093/aje/kwj169 16675535

[43] Sariaslan A, Fazel S, D’Onofrio BM, Schizophrenia and subsequent neighborhood deprivation: revisiting the social drift hypothesis using population, twin and molecular genetic data. Transl Psychiatry. 2016;6(5):e796-e796. doi:10.1038/tp.2016.62 27138795 PMC5070045

