# Supplementary material for: Assessment of the Role of IQ in Associations Between Population Density and Deprivation and Nonaffective Psychosis
Source: JAMA Psychiatry. 2020 Mar 11;77(7):1–8. doi: 10.1001/jamapsychiatry.2020.0103 (PMC7066518; doi:10.1001/jamapsychiatry.2020.0103)
Supplement: Supplement. — eFigure 1. Distribution of Population Density Scores at Birth in the Sample With Complete Data Used for Analyses (n = 227 429) eFigure 2. Flow of Participants Through the Study eFigure 3. Mediation Model for the Association Between Population Density/Deprivation and Nonaffective Psychosis eMethods 1. Measures and Statistical Analysis eMethods 2. Mediation eMethods 3. Auxiliary Variables Included in Multiple Imputation Models eMethods 4. Power Simulation of Effect Modification eTable 1. Baseline Characteristics of Samples With Complete and Missing Data eTable 2. Univariable and Multivariable Odds Ratios (OR) for Associations Among Population Density, Deprivation, IQ, and Nonaffective Psychosis, Excluding Those Diagnosed Within 2 Years of Conscription (n = 142) eTable 3. Univariable and Multivariable Odds Ratios (OR) for Associations Between Population Density, Deprivation, IQ, and Nonaffective Psychosis, Further Adjusted for Paternal IQ eTable 4. Univariable and Multivariable Change in IQ According to Population Density and Deprivation Further Adjusted for Paternal IQ eTable 5. Univariable and Multivariable Change in IQ According to Population Density and Deprivation Multiply Imputed Sample and Further Adjusted for Paternal IQ eTable 6. Univariable and Multivariable Odds Ratios (OR) for Associations Between Population Density, Deprivation, IQ, and Nonaffective Psychosis in the Sample With Missing Data Replaced by Multiple Imputation eTable 7. Univariable and Multivariable Change in IQ Score According to Population Density and Deprivation in the Sample With Missing Data Replaced by Multiple Imputation eTable 8. List of Diagnostic Codes for Obstetric Complications and Early Life (0-12 Months) Infections as Recorded in the Maternal Birth Register eTable 9. List of Diagnostic Codes for Early Life (0-12 Months) Infections as Recorded in the National Patient Register eTable 10. Univariable and Multivariable Change in IQ Score According to Population Density and Deprivation [file jamapsychiatry-77-729-s001.pdf]

Supplementary Online Content

Lewis G, Dykxhoorn J, Karlsson H, et al. Assessment of the role of IQ in associations between population density and deprivation and nonaffective psychosis. Published online March 11, 2020. *JAMA Psychiatry.*
doi:10.1001/jamapsychiatry.2020.0103

eFigure 1. Distribution of Population Density Scores at Birth in the Sample With Complete Data Used for Analyses (n = 227 429)
eFigure 2. Flow of Participants Through the Study
eFigure 3. Mediation Model for the Association Between Population Density/Deprivation and Nonaffective Psychosis
eMethods 1. Measures and Statistical Analysis
eMethods 2. Mediation
eMethods 3. Auxiliary Variables Included in Multiple Imputation Models
eMethods 4. Power Simulation of Effect Modification
eTable 1. Baseline Characteristics of Samples With Complete and Missing Data
eTable 2. Univariable and Multivariable Odds Ratios (OR) for Associations Among Population Density, Deprivation, IQ, and Nonaffective Psychosis, Excluding Those Diagnosed Within 2 Years of Conscription (n = 142)
eTable 3. Univariable and Multivariable Odds Ratios (OR) for Associations Between Population Density, Deprivation, IQ, and Nonaffective Psychosis, Further Adjusted for Paternal IQ
eTable 4. Univariable and Multivariable Change in IQ According to Population Density and Deprivation Further Adjusted for Paternal IQ
eTable 5. Univariable and Multivariable Change in IQ According to Population Density and Deprivation Multiply Imputed Sample and Further Adjusted for Paternal IQ
eTable 6. Univariable and Multivariable Odds Ratios (OR) for Associations Between Population Density, Deprivation, IQ, and Nonaffective Psychosis in the Sample With Missing Data Replaced by Multiple Imputation
eTable 7. Univariable and Multivariable Change in IQ Score According to Population Density and Deprivation in the Sample With Missing Data Replaced by Multiple Imputation
eTable 8. List of Diagnostic Codes for Obstetric Complications and Early Life (0-12 Months) Infections as Recorded in the Maternal Birth Register
eTable 9. List of Diagnostic Codes for Early Life (0-12 Months) Infections as Recorded in the National Patient Register
eTable 10. Univariable and Multivariable Change in IQ Score According to Population Density and Deprivation, Excluding Those Diagnosed With Nonaffective Psychosis Within 2 years of Conscription (n = 142)
eTable 11. Univariable and Multivariable Odds Ratios (OR) for Associations Between Population Density, Deprivation, IQ, and Nonaffective Psychosis, Multiply Imputed Sample and Further Adjusted for Paternal IQ
eTable 12. Power Simulations for Interaction Between Population Density and Deprivation at Birth and IQ on Risk of Nonaffective Psychosis for Various Hypothesized Effect Sizes
eReferences.

This supplementary material has been provided by the authors to give readers additional information about their work.

© 2020 Lewis G et al. *JAMA Psychiatry.*

eFigure 1. Distribution of Population Density Scores at Birth in the Sample With Complete Data Used for Analyses (n = 227 429)

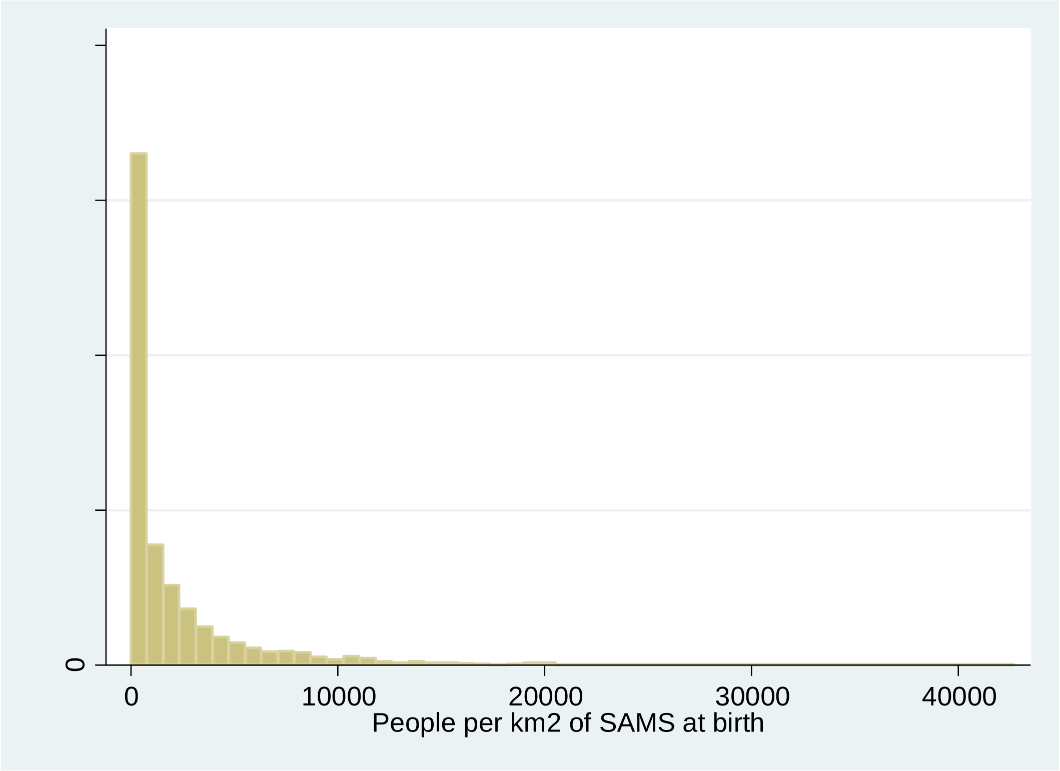

© 2020 Lewis G et al. *JAMA Psychiatry.*

eFigure 2. Flow of Participants Through the Study

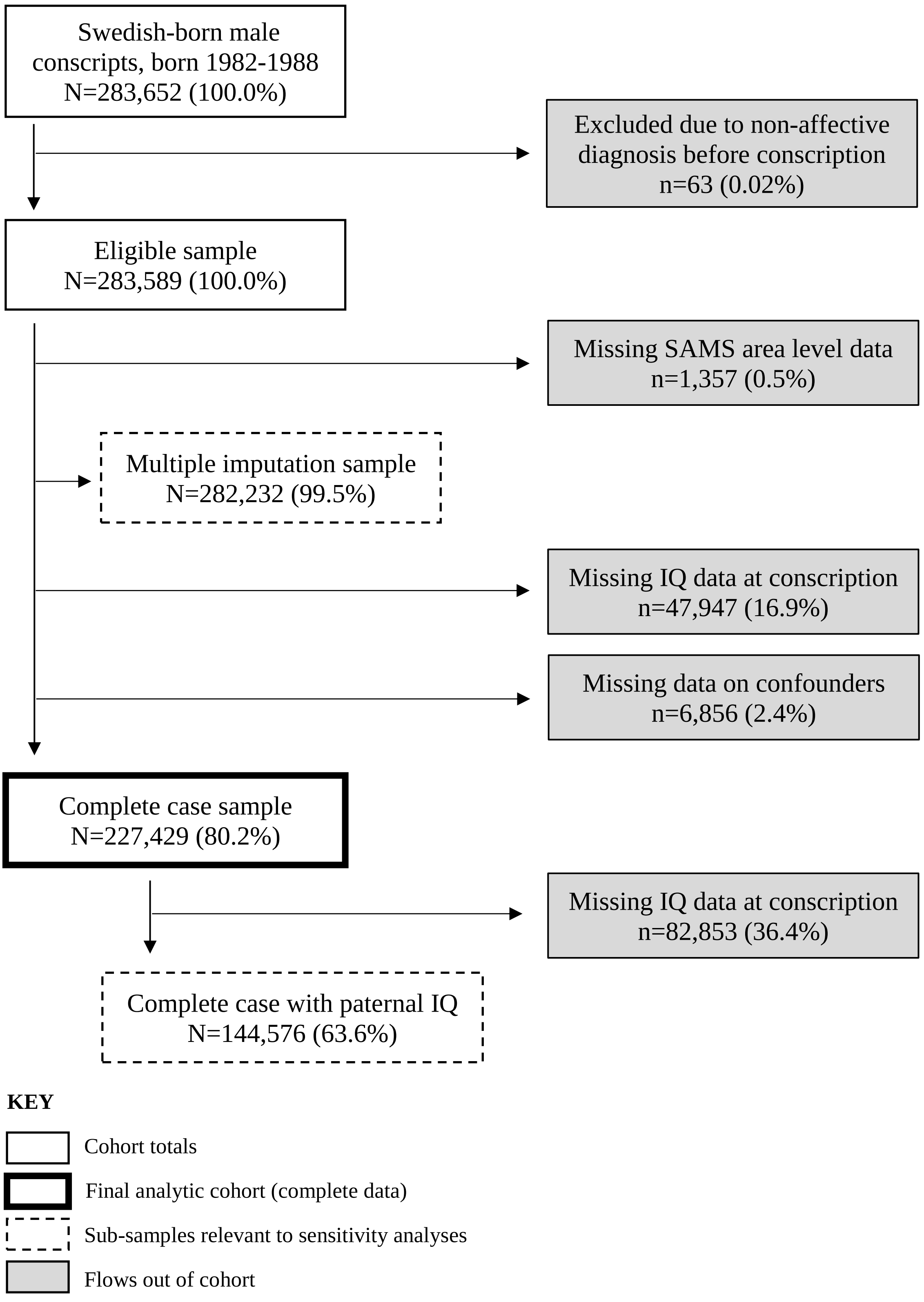

SAMS: Small Area Marketing Statistics

© 2020 Lewis G et al. *JAMA Psychiatry.*

eFigure 3. Mediation Model for the Association Between Population Density/Deprivation and Nonaffective Psychosis

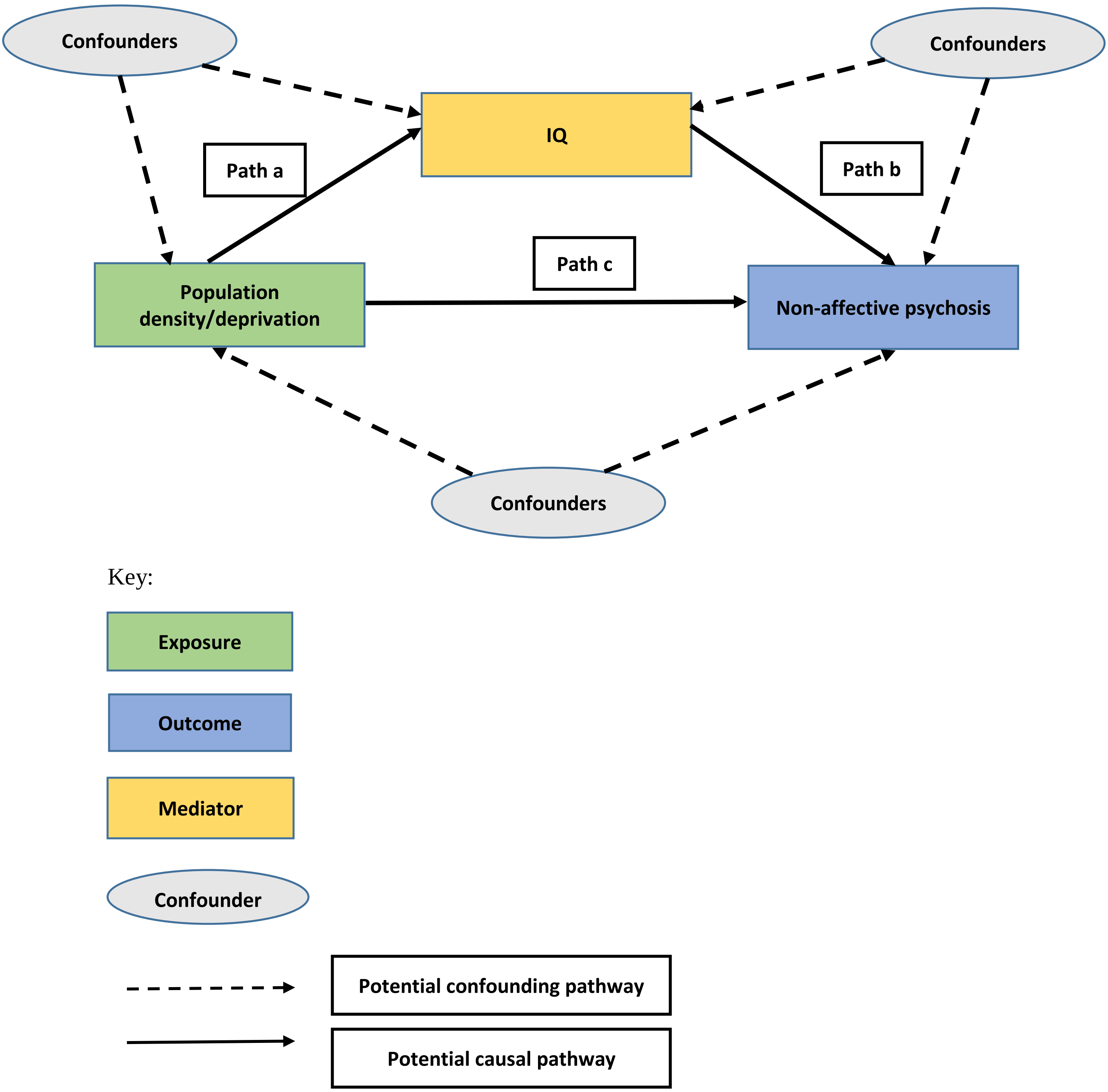

© 2020 Lewis G et al. *JAMA Psychiatry.*

eMethods 1. Measures

IQ

The IQ tests for Swedish conscripts have been described in detail previously.[1] The Swedish military service conscription examination involves a medical assessment, including general intelligence, with IQ scores summed using four scales to assess verbal, logical, spatial, and technical abilities. Four tests were done measuring verbal, logical, spatial, and technical abilities. Each test gave a score ranging from 1-9 and these tests were summed to give a total general intelligence score.

The verbal IQ test lasted seven minutes and had 40 questions. Participants were given rows of five words and told to underline the odd one out.

The spatial test took 40 minutes. Participants were shown a focal geometric shape. They were then four other shapes of different sizes and orientations and asked which of these shapes could be used to form the focal geometric shape they had originally seen.

The logic test comprised tests of general knowledge and intelligence and had 40 items and took 12 minutes. Participants were presented with a combination of shapes and letters. They were asked questions such as "put a line through the square under the longest word." There were also asked questions on general knowledge and mathematics.

The technical test required a basic knowledge of physics and mechanical ability. For example, "a 100 kg bail of hay and 100 kg of sand both resting on a sharp incline. The question was which would tip over more easily"?[1]

Deprivation

© 2020 Lewis G et al. *JAMA Psychiatry.*

We estimated deprivation for each participant's SAMS region in their birth year based on a composite measure of levels of crime, unemployment, low income and receipt of social benefits (see Supplement). with a criminal conviction (from the National Register of Criminal Convictions), who received social benefit, who were unemployed, and who had income below the national median (from the Longitudinal Integration Database for Health Insurance and Labour Market Studies [LISA]). Each indicator was z-standardized and summed to derive overall deprivation, greater scores indicating more deprived areas.

Confounders

We obtained potential confounder information by linking our participants to other relevant registers (Register of the Total Population, Multigeneration register, National Patient Register (NPR), Maternal Birth Register (MBR), National Schools Register; the Longitudinal Integration Database for Health Insurance and Labour Market Studies [LISA]).

We adjusted for paternal age given prior evidence of a strong positive association with offspring psychosis,[2] and theoretical grounds that it may be associated with our exposures. We used a binary variable for parental history of severe mental illness, which was coded 1 if either parent had ever been diagnosed with non-affective psychosis or bipolar disorder since 1973, when the National Patient Register began to collect data on psychiatric admissions (ICD-9 codes: 295.x, 296.x; ICD-10 codes: F20-31).[3]

Parental time in education used the following categories: (less than 9 years compulsory education; 9 years compulsory education; secondary education; post-secondary education for less than 2 years; post-secondary education for 2+ years; doctorate education)

© 2020 Lewis G et al. *JAMA Psychiatry.*

Family disposable income when the offspring was born was based on information in the LISA on total family income including wages, welfare benefits, other social subsidies, and pensions. We categorized family disposable income into quintiles, relative to all other people in Sweden in the given year to account for inflation.[4]

Migrant status (Swedish-born to two Swedish-born parents, or Swedish-born to at least one foreign-born parent) was coded from the Register of the Total population and the Multigeneration register.

For each participant we obtained their parents time in education as recorded in the National School Register, and classified this as less than 9 years compulsory education, 9 years compulsory education, secondary education, post-secondary education for less than 2 years, post-secondary education for 2+ years or doctorate education. Where available, paternal IQ (from earlier conscript data) was also recorded.

Missing data

To establish whether missing data were associated with our exposures and outcome (and might therefore introduce bias), we compared the characteristics of those with and without complete data. We also conducted a sensitivity analysis using multiple imputation with chained equations (MICE). We assumed data were missing at random and imputed 20 datasets, combined using Rubin's rules. To impute missing data, we used conscripts' educational attainment at age 16, all characteristics described and several auxiliary variables (maternal smoking, obstetric complications and infections in the first year of the participants' life[22] and childhood residential mobility[27]; see Supplemental Tables 8-9).

© 2020 Lewis G et al. *JAMA Psychiatry.*

Statistical analyses

Associations of population density and deprivation with non-affective psychosis

We used multilevel logistic regression with individuals (level 1) clustered in SAMS regions (level 2), and a random intercept at the SAMS level. First, we ran separate univariable models for associations between each exposure and non-affective psychosis. Second, we ran a bivariable model including population density and deprivation. We then adjusted this model for potential confounders.

Population attributable fraction

We calculated the population attributable fraction (PAF) for the main exposures, deprivation and population density. In reality these exposure are continuous and it would be arbitrary to create a category for whether people were exposed or unexposed. Consistent with a previous study, we therefore considered this a theoretical exercise and calculated the PAF as an estimate of the proportion of non-affective psychoses that could be prevented if we could identify and remove all factors that lead to increased incidence associated with deprivation and population density.[3]

Modification by IQ

We tested for interactions first, so that any interaction terms significant at an alpha level of <.05 could be modelled in subsequent mediation analyses. We first added conscript IQ to the fully-adjusted model described above. Next, we sequentially fitted two interaction terms to test whether the associations between population density and deprivation at birth respectively, and subsequent risk of non-affective psychosis, were modified by IQ. Effect modification was assessed via likelihood ratio test (LRT). We conducted power analysis via simulations of

© 2020 Lewis G et al. *JAMA Psychiatry.*

these two interactions on non-affective psychosis, based on our analytic sample size for the (i) observed interaction effect sizes (odds ratios (OR)), and ORs of (ii) 1.10 and (iii) 1.20 (see Supplemental Materials for full details).

Mediation by IQ

Figure 1 shows our hypothesized mediation model. To determine whether population density or deprivation at birth were associated with IQ scores at age 18 years (Path A, Figure 1) we used multilevel linear regression. We tested whether IQ was associated with non-affective psychosis (Path B, Figure 1) using multilevel logistic regressions. Path C was assessed above. All models were run before and after adjusting for confounders. including paternal IQ in a sensitivity analysis. We formally tested for mediation using the “potential outcomes” framework (see below),[32] a class of causal mediation analysis fitted using parametric mediation models.[30,31]

Sensitivity analyses

Consistent with a previous study[22] and to reduce the possibility that IQ at age 18 captured a prodromal effect of non-affective psychosis, we re-ran analyses of the association between IQ and non-affective psychosis after excluding participants diagnosed with non-affective psychosis within two years of conscription. We also conducted sensitivity analyses to additionally adjust for paternal IQ, which was only available on a subset of participants.. Sensitivity analyses with missing data replaced by multiple imputation were reported for all associations except the causal mediation analysis, since multiple imputation approaches in this context are not yet routinely available. All analyses were done in Stata version 15.

eMethods 2. Mediation

We fitted parametric mediation models[9,10] using the STATA command ‘paramed’ to investigate the potentially mediating role of IQ on the associations between population density and deprivation at birth and risk of non-affective psychosis. Traditional mediation models make the assumption of no exposure (A) – mediator (M) interaction, and in the presence of such interaction, direct and indirect effect estimates will be biased, making effect decomposition unreliable. Further, traditional mediation approaches are only valid when the mediator and outcome (Y) are treated as continuous outcomes under linear regression. Both traditional and causal mediation analyses assume that the models are correctly specified, with no unobserved confounding between M and Y.

By adopting a counterfactual – or potential outcomes – approach we can potentially overcome these limitations to produce unbiased effect decomposition into total, controlled direct and natural indirect effects, provided that four main assumptions are satisfied:

1. No unmeasured A>Y confounding
2. No unmeasured M>Y confounding
3. No unmeasured A>M confounding
4. No M>Y confounder that is caused by A

The potential outcomes framework thus makes the (strong) assumption of ‘conditional exchangeability’[11]; that individuals who are exposed or unexposed (or across different levels of the mediator) are exchangeable, having conditioned on all confounders; it is, of course, difficult to be certain that all confounders have been identified and precisely measured using observational data. Theoretically, the potential outcomes framework tests causal assumptions in the hypothetical circumstances of everyone being either exposed or unexposed. In our

© 2020 Lewis G et al. *JAMA Psychiatry.*

study, we calculate the 'total effect' of population density/deprivation on non-affective psychosis, which comprises a 'direct effect' and an 'indirect effect' (the latter is an estimate of how much of the association between population density/deprivation and non-affective psychosis is mediated by IQ). The total effect estimates the 'average causal effect (ACE)' or change in outcome status if everyone moved from unexposed to exposed ($E\{Y(1) - Y(0)\}$). We estimated the 'controlled' direct effect, which compares outcomes under exposure levels $A=1$ versus $A=0$, fixing $M=M(0)$, where A=exposure and M=mediator. By fixing M to 0, the controlled direct effect estimates what the association between exposure and outcome would be if the mediator had the same value in those exposed and unexposed (i.e. if changes in the mediator were not induced by exposure). The natural indirect effect compares outcomes under the counterfactual scenarios where $M=M(1)$ versus $M=M(0)$, fixing $A=1$ (everyone is exposed, and the outcome is compared between those who do and do not experience the mediator). We expressed indirect effects as a percentage of the total effect by log transforming the odds ratios and dividing the indirect effect by the total effect.

eMethods 3. Auxiliary Variables Included in Multiple Imputation Models

We obtained data on maternal smoking during pregnancy, obstetric complications (OC) and early life infections in infancy (0-12 months) from the MBR and NPR. Maternal smoking during pregnancy was recorded at the first antenatal visit and recoded as "none", "1-9 cigarettes per day" or "10 or more cigarettes per day". We considered the following major obstetric complications, based on evidence from a previous systematic review that they were associated with future risk of schizophrenia,[5] or evidence that they were associated with childhood cognitive function[6]: maternal diabetes (yes/no), non-elective Caesarian section (yes/no), any maternal hypertension disorders during pregnancy (including pre-eclampsia) (yes/no), small for gestational age, 1 minute Apgar score less than 7 (rated 0-10), asphyxia

© 2020 Lewis G et al. *JAMA Psychiatry.*

(yes/no) and congenital malformations (yes/no). Full details and ICD codes are given in Supplemental Table 8. We identified early life infections resulting in hospitalization in the first year of life, as recorded in the NPR. We included the same set of infections as used in a major previous investigation from our group (see Supplemental Table 9 for ICD codes),[7] and coded participants according to the number of times they received a diagnosis for one of these infections in the NPR, categorized as 0, 1, 2 or 3+ diagnoses. We also included the number of small area (SAMS) residential moves in childhood and adolescence as auxiliary data, which has been previously associated with risk of non-affective psychosis in this sample, and may inform missing covariate data in this study. Full details of the derivation of these variables are described elsewhere,[4] but briefly, we calculated the number of times a participant moved to a different SAMS area from one year to the next, using data provided in the Register of the Total Population. Separate variables were estimated for moves between 0-6, 7-15 and 16-19 years old. Finally, we included participant scholastic achievement in final exams at the end of compulsory education (~16 years old) as an auxiliary variable to predict missing IQ and covariate data, categorized as “A/B”, “C”, “D/E”, fail or missing. Poor scholastic achievement has previously been associated with greater risk of psychotic disorders in Swedish register data.[8]

eMethods 4. Power Simulation of Effect Modification

Power analysis via simulation was conducted for effect modification between population density at birth and IQ, and deprivation at birth and IQ, simultaneously on risk of non-affective psychosis. Simulations were based on our analytic sample size (N=227,410) and power calculations were estimated for the (i) observed interaction effect sizes (for both population density by IQ, and deprivation by IQ, observed odds ratios were 1.01; 95%CI: 0.97 to 1.06), and effect sizes (odds ratios) of (ii) 1.05 and (iii) 1.10. Simulations accounted

© 2020 Lewis G et al. *JAMA Psychiatry.*

for correlations between population density, deprivation and IQ and were fitted in a generalized linear model with a binomial distribution and logit link function. Alpha was set to 0.05 and 500 Monte Carlo replications per simulation. Power simulations were fitted using the user-written *powersim* command in Stata, based on the full methodology described by Luedicke.[12]

While we were underpowered to detect interaction effects of the magnitudes observed (Supplemental Table 12), we had over 98% power to detect interaction odds ratios greater than 1.05 and 100% power for effect sizes greater than 1.10 (Supplemental Table 12).

© 2020 Lewis G et al. *JAMA Psychiatry.*

eTable 1. Baseline Characteristics of Samples With Complete and Missing Data

Characteristic	Missing data (n=56,160)[a]	Complete data (227,429)[b]	P value[c]
Most urban population density category	10,131 (18.4)	34,664 (15.2)	<.0001
Most deprived category	6608 (12.2)	22,340 (9.8)	<.0001
Non-affective psychosis	599 (1.1)	1596 (0.7)	<.0001
Maternal compulsory education less than 9 years (lowest category)	2094 (3.9)	6905 (3.0)	<.0001
Paternal compulsory education less than 9 years (lowest category)	3903 (7.8)	15,427 (6.8)	<.0001
Lowest family income quintile	2295 (4.1)	5900 (2.6)	<.0001
History of any psychosis in either parent	1987 (3.6)	6395 (2.8)	<.0001
Parents migrated into Sweden	11,380 (20.3)	31,338 (13.8)	<.0001
IQ score (mean and SD)	97.04 (15.80)	99.96 (15.29)	<.0001
Mother age at birth, years (mean and SD)	28.39 (5.22)	28.47 (5.01)	.001
Father age at birth, years (mean and SD)	31.44 (6.08)	31.30 (5.73)	<.0001

[a] Excluded from analyses because of missing data on IQ or other variables (20%).
[b] Sample containing complete data on exposures, outcome, IQ and all potential confounders
[c] P-values compare those with missing data and those without and for categorical characteristics are drawn from chi-squared tests and for continuous characteristics from linear regressions.
Data are n (%) unless otherwise specified.

© 2020 Lewis G et al. *JAMA Psychiatry.*

eTable 2. Univariable and Multivariable Odds Ratios (OR) for Associations Among Population Density, Deprivation, IQ, and Nonaffective Psychosis, Excluding Those Diagnosed Within 2 Years of Conscription (n = 142)

Exposure variable	Non-affective psychosis (n= 227,287)											
	Unadjusted[d]			Bivariable[e]			Multivariable[f]			Fully-adjusted[g]		
	OR	95% CI	p	OR	95% CI	p	OR	95% CI	p	OR	95% CI	p
**Population density[a]**	1.17	1.12 to 1.22	<.0001	1.14	1.09 to 1.19	<.0001	1.10	1.05 to 1.15	<.0001	1.10	1.05 to 1.15	<.0001
**Deprivation[b]**	1.17	1.12 to 1.23	<.0001	1.13	1.08 to 1.18	<.0001	1.07	1.02 to 1.12	.011	1.05	1.00 to 1.10	.073
**IQ[c]**	.72	.68 to .75	<.0001	-	-	-	-	-	-	.71	.67 to .75	<.0001

[a] Units are number of people per km^2 and unit change is per one standard deviation (3863.97 people per km^2)
[b] Units are based on the deprivation index and unit change is per one standard deviation (1.96 points)
[c] Units are based on the Wechsler Adult Intelligence Scale unit change is per one standard deviation (15 points)
[d] Separate univariable models for population density, deprivation and IQ
[e] Bivariable model for population density and deprivation
[f] Population density and deprivation adjusted for paternal age at birth, family income, maternal education, paternal education, any psychosis or bipolar in parents, parent/s born outside of Sweden.
[g] The above multivariable model, also with IQ

© 2020 Lewis G et al. *JAMA Psychiatry.*

eTable 3. Univariable and Multivariable Odds Ratios (OR) for Associations Between Population Density, Deprivation, IQ, and Nonaffective Psychosis, Further Adjusted for Paternal IQ

Exposure variable	Non-affective psychosis (n=144,576)											
	Unadjusted[d]			Bivariable[e]			Multivariable[f]			Fully-adjusted[g]		
	OR	95% CI	p	OR	95% CI	p	OR	95% CI	p	OR	95% CI	p
**Population density[a]**	1.17	1.12 to 1.21	<.0001	1.13	1.09 to 1.18	<.0001	1.07	1.02 to 1.13	.008	1.10	1.03 to 1.16	.002
**Deprivation[b]**	1.18	1.12 to 1.23	<.0001	1.13	1.08 to 1.19	<.0001	1.10	1.04 to 1.16	.002	1.05	1.01 to 1.15	.025
**IQ[c]**	.71	.68 to .75	<.0001	-	-	-	-	-	-	.73	.69 to .78	<.0001

[a] Units are number of people per km^2 and unit change is per one standard deviation (3863.97 people per km^2)
[b] Units are based on the deprivation index and unit change is per one standard deviation (1.96 points)
[c] Units are based on the Wechsler Adult Intelligence Scale unit change is per one standard deviation (15 points)
[d] Separate univariable models for population density, deprivation and IQ
[e] Bivariable model for population density and deprivation
[f] Population density and deprivation adjusted for paternal age at birth, family income, maternal education, paternal education, any psychosis or bipolar in parents, parent/s born outside of Sweden and paternal IQ.
[g] The above multivariable model, also with IQ

© 2020 Lewis G et al. *JAMA Psychiatry.*

eTable 4. Univariable and Multivariable Change in IQ According to Population Density and Deprivation Further Adjusted for Paternal IQ

Exposure variable	IQ score (n=144,576)								
	Unadjusted[c]			Bivariable[d]			Fully-adjusted[e]		
	Change in IQ score	95% CI	p	Change in IQ score	95% CI	p	Change in IQ score	95% CI	p
**Population density[a]**	.15	.03 to .26	.014	.61	.50 to .72	<.0001	.05	-.03 to .13	.223
**Deprivation[b]**	-1.58	-1.68 to -1.48	<.0001	-1.72	-1.82 to -1.62	<.0001	-.28	-.36 to -.20	<.0001

Path A of Mediation Model, eFigure 1

[a] Units are number of people per km^2 and unit change is per one standard deviation

[b] Units are based on the deprivation index and unit change is per one standard deviation

[c] Separate univariable models for population density and deprivation

[d] Bivariable model for population density and deprivation

[e] Population density and deprivation adjusted for paternal age at birth, family income, maternal education, paternal education, any psychosis or bipolar in parents, parent/s born outside of Sweden and paternal IQ.

© 2020 Lewis G et al. *JAMA Psychiatry.*

eTable 5. Univariable and Multivariable Change in IQ According to Population Density and Deprivation Multiply Imputed Sample and Further Adjusted for Paternal IQ

Exposure variable	IQ score (n=282,232)								
	Unadjusted[c]			Bivariable[d]			Fully-adjusted[e]		
	Change in IQ score	95% CI	p	Change in IQ score	95% CI	p	Change in IQ score	95% CI	p
**Population density[a]**	.12	.010 to .23	.033	.61	.51 to .72	<.0001	-.01	-.10 to .07	.073
**Deprivation[b]**	-1.61	-1.70 to -1.51	<.0001	-1.76	-1.85 to -1.66	<.0001	-.44	-.52 to -.36	<.0001

Path A of mediation model, eFigure 1
[a] Units are number of people per km^2 and unit change is per one standard deviation
[b] Units are based on the deprivation index and unit change is per one standard deviation
[c] Separate univariable models for population density and deprivation
[d] Bivariable model for population density and deprivation
[e] Population density and deprivation adjusted for paternal age at birth, family income, maternal education, paternal education, any psychosis or bipolar in parents, parent/s born outside of Sweden and paternal IQ.

© 2020 Lewis G et al. *JAMA Psychiatry.*

eTable 6. Univariable and Multivariable Odds Ratios (OR) for Associations Between Population Density, Deprivation, IQ, and Nonaffective Psychosis in the Sample With Missing Data Replaced by Multiple Imputation

Exposure variable	Non-affective psychosis (n=282,232)											
	Unadjusted[d]			Bivariable[e]			Multivariable[f]			Fully-adjusted[g]		
	OR	95% CI	p	OR	95% CI	P	OR	95% CI	p	OR	95% CI	p
**Population density[a]**	1.17	1.13 to 1.21	<.0001	1.13	1.09 to 1.17	<.0001	1.08	1.04 to 1.13	.008	1.08	1.05 to 1.12	<.0001
**Deprivation[b]**	1.18	1.14 to 1.23	<.0001	1.14	1.09 to 1.18	<.0001	1.08	1.04 to 1.13	.002	1.06	1.02 to 1.11	.005
**IQ[c]**	0.73	0.69 to 0.76	<.0001	-	-	-	-	-	-	0.71	0.68 to 0.75	<.0001

[a] Units are number of people per km^2 and unit change is per one standard deviation (3863.97 people per km^2)
[b] Units are based on the deprivation index and unit change is per one standard deviation
[c] Units are based on the Wechsler Adult Intelligence Scale unit change is per one standard deviation
[d] Separate univariable models for population density, deprivation and IQ
[e] Bivariable model for population density and deprivation
[f] Population density and deprivation adjusted for paternal age at birth, family income, maternal education, paternal education, any psychosis or bipolar in parents, parent/s born outside of Sweden.
[g] The above multivariable model, also with IQ. This model was then run again with interaction terms for population density and IQ and deprivation and IQ.

© 2020 Lewis G et al. *JAMA Psychiatry.*

eTable 7. Univariable and Multivariable Change in IQ Score According to Population Density and Deprivation in the Sample With Missing Data Replaced by Multiple Imputation

Exposure variable	IQ score (n=282,232)								
	Unadjusted[c]			Bivariable[d]			Fully adjusted[e]		
	Change in IQ score	95% CI	p	Change in IQ score	95% CI	p	Change in IQ score	95% CI	p
**Population density[a]**	.12	.010 to .23	.033	.61	.51 to .72	<.0001	.07	-.01 to .15	.091
**Deprivation[b]**	-1.61	-1.70 to -1.51	<.0001	-1.76	-1.85 to -1.66	<.0001	-.73	-.81 to -.65	<.0001

Path A of mediation model, eFigure 2
[a] Units are number of people per km^2 and unit change is per one standard deviation
[b] Units are based on the deprivation index and unit change is per one standard deviation
[c] Separate univariable models for population density and deprivation
[d] Bivariable model for population density and deprivation
[e] Population density and deprivation adjusted for paternal age at birth, family income, maternal education, paternal education, any psychosis or bipolar in parents, parent/s born outside of Sweden.

© 2020 Lewis G et al. *JAMA Psychiatry.*

eTable 8. List of Diagnostic Codes for Obstetric Complications and Early Life (0-12 Months) Infections as Recorded in the Maternal Birth Register

**Variable**	**ICD-10 codes**	**ICD-9 codes**	**ICD-8 codes**	**Notes**
Maternal diabetes	E10-E11	250	250	Diabetes Miletus
Non-elective C-section	O82.1			
Maternal hypertension disorders in pregnancy	O10, O14, O15	642	63701, 63703, 63704, 63709, 63710, 63799	
Asphyxia	P200-P229	768C-G. X 769A-B, X	77610, 77629, 77640, 77650	
Congenital malformations	Q00-Q99	740-759	740-759	
1-minute Apgar score	-	-	-	As recorded in the MBR
Small for gestational age	-	-	-	Only available for singleton births, as recorded in MBR

© 2020 Lewis G et al. *JAMA Psychiatry.*

eTable 9. List of Diagnostic Codes for Early Life (0-12 Months) Infections as Recorded in the National Patient Register[a]

Infection type	ICD Version[b]	Diagnostic codes
Any infection	ICD-8	006.00-007.99, 009.00-009.98, 084.00-087.99, 099.96-099.99, 110.00- 130.10, 130.99-131.99, 136.09, 320.88-320.99, 360.00, 380.02-381.99, 384.00-384.08, 420.00-420.09, 421.98, 422.97-422.99, 462.01, 462.09, 463.09, 466.99, 483.99-486.09, 503.00-503.09, 540.00-540.02, 540.04- 540.99, 572,99, 686.00-686.98, 761,40, 763.10, 763.98, 778,60 + ICD-8 codes in 'Bacterial infection' and 'Viral infection'.
	ICD-9	006-007X, 008W, 009-D, 084-086X, 099E-X, 110-136X, 321A, 321W, 370E-F, X, 372A-D, 380B, C, 381A, 382X, 420- 422X, 462-463, 466-B, 473-X, 483, 485-486, 490, 491B, 540A, X, 572A, 647C, E, W, X, 680A, 711G-X, 727A, 770A, 771C, E-W + ICD-9 codes in 'Bacterial infection' and 'Viral infection'.
	ICD-10	A06-07.9, A08.5, A09, A59-59.9, A63, A63.8-64, B35-49, B50 -89, B99, G02.1-02.8, G04, G04.9, G05.2, H10.0, H10.3-10.9, H16.2-16.3, H16.9, H32, H60, H60.3, H65.0-65.1, H66.9, I30.0-30.9, I33.0-33.9, I40.0, J02*, J02.8-02.9*, J03*, J03.8-03.9*, J16, J16.8, J18-18.9, J20, J20.8-21, J21.8- 21.9, J22, J32-32.9*, J35.0, J37-37.1*, J40-42, K35, K35.9, K75.0, L30.3, M46.5, M65.1, M71.1, O98.3, O98.6-98.9, P23.8-23.9, P37.1-39.9, Z22.4, Z22.8-22.9 + ICD-10 codes in 'Bacterial infection' and 'Viral infection'.
Bacterial	ICD-8	000.01-005.99, 008.00-008.30, 010.99-018.98, 020.00-039.98, 073.99, 076.99, 079.30, 080.99-083.99, 088.99-104.98, 320.00-320.80, 322.00- 322.03, 361.00-361.09, 362.02, 366.00, 369.00, 380.00-380.01, 382.00- 383.99, 390.97-392.99, 421.00, 461.00-461.09, 462.02, 463.01, 481,99- 482.98, 501.99, 508.00-508.02, 510.01-510.09, 511.10, 513.99, 522.50, 527.30, 528.00, 528.30, 540.03, 562.00-562.19, 566.00-566.01, 567.00- 567.02, 569.00, 577.01, 590.00-590.99, 595.00-595.02, 597.00, 599.02, 611.00, 611.01, 612.01-614.99, 616.00-616.03, 620.00-620.99, 622.00- 622.19, 629.40, 630.00-630.09, 635.00-636.09, 645.90-645.91, 670.00- 670.09, 678.02, 680.00-682.99, 684.00-684.09, 710.00-710.09, 720.00-720.29, 732.99, 761,00, 763.00, 998.50, 999.30
	ICD-9	001-005X, 008A-F, 010-041X, 073, 076, 078D, J, 790H, 080-083X, 087- 099D, 100-104, 245A, 254B, 320-X, 324-X, 360A, 373B, 375D, 376A, 382A-E, 383A-X, 390-392X, 421A, 461-X, 475, 481-482X, 510-X, 511B, 513-B, 522E, H, 526E, 527D, 528A, D, 540B, 562-B, 566, 567-C, 569F, 575A, 590-X, 597A, 595-D, X, 597W, 599A, 611A, 614-F, W-X, 615A, X, 616-X, 634A, 635A, 636A, 637A, 638A, 639A, 646F, G, 647A, B, D, 658E, 659D, 670, 675-B, W-X, 681-686X, 711A, E, 728A, 729E, 730-D, X, 771D, 996G, 998F, 999D
	ICD-10	A00-05.9, A15-17.9, A20-28.9, A30 -58, A65 -79.9, B95-96.8, E06.0, E32.1, G00-00.9, G01, G04.2, G05.0, G06-06.2*, G07, H00.0, H01.0, H04.3, H05.0, H44.0, H60.0-60.1, H66.0-66.4, H70.0-70.9, I00-02.9, J01-01.9*, J02.0, J03.0, J13-15.9, J16.0, J20.0-20.2, J34.0, J36*, J39.0-39.1, J85.1-85.3, J86- 86.9*, K04.6-04.7, K05.2, K11.3, K12.2, K14.0, K35.1, K57-57.9, K61-61.4, K63.0, K65.0*, K81.0, K85, L00 -08.9, M00-00.9, M46.3*, M60.0*, M86- 86.9*, N10-12*, N13.6*, N15.1, N15.9, N30-30.3*, N30.8-30.9*, N34-34.1*, N39.0*, , N61, N70-76.8*, N98.0, O07.0, O07.5, O08.0, O23-23.9, O41.1, O75.3, O85-86.8*, O91-91.1, O98.0-98.2, P23.1-23.6, P36, P37.0, T80.2, T81.4, T82.6-82.7, T83.5-83.6, T84.5-84.7, T85.7, T88.0, Z22.0-22.3
Viral	ICD-8	008.80-008.98, 040.00-043.99, 045.00-065.99, 067.00-072.09, 074.00- 075.09, 078.00-079.20, 079.40-079.99, 099.92, 460.99, 464.01-480,99, 508.03, 761.20, 761.30

© 2020 Lewis G et al. *JAMA Psychiatry.*

	ICD-9	008H-M, 045-066, 070-072X, 074-075, 077-078H, 078W-079X, 279K, 321B-H, 323A, 323C-D, 460, 464-465X, 480-X, 487-W, 647F, G, 711F, 771A, B, 790W
	ICD-10	A08-08.4, A60-60.9, A63.0, A80-89, A90-99, B00-06.0, B06.8-09, B15- 19.9, B20-24, B25-34, B97-97.8, G02.0, G05.1, J00, J04-06.9*, J10-11.8, J12-12.9, J20.3-20.7, J21.0, O35.3, O98.4-98.5, P23.0, P35, Z21, Z22.5-22.6
CNS	ICD-8	013.00-013.99, 027.01, 036.00, 090.40, 094.00-094.98, 320.00-320.80, 322.00-322.03, 392.99, 040.00-043.99, 045.00-046.99, 052.00, 054.04, 062.00-065.99, 071.99, 072.01, 075.02, 079.20, 474.99, 084.00, 320.88- 320.99
	ICD-9	013-X, 036A, B, 090E, 094-X, 320-X, 324-X, 392-X, 045-049X, 054D, 052B, 053A, 055A, 056A, 071, 072B, C, 321B-H, 323A, 323C, D, 006F, 321A, 321W
	ICD-10	A02.2 (if G01), A17-17.9, A20.3, A22.8, A32.1, A39.0, A39.8 (if G05), A50.4 (if G05.0 or G01), A51.4 (if G01), A52.1 (if G05.0, G01 or F02.8), A54.8 (if G07 or G01), A69.2 (if G01), G00-00.9, G01, G04.2, G05.0, G06- 06.2, G07, I02-02.9, A80-89, B00.3-00.4, B01.0-01.1, B02.0-02.1, B05.0- 05.1, B06.0, B26.1-26.2, G02.0, G05.1, B58.2, A06.6, B37.5, B38.4, B43.1, B45.1, B46.1, B50.0, B57.4, B60.2, B69.0, B83.2, G02.1-02.8, G04, G04.9, G05.2
Respiratory	ICD-8	010-012, 020.10, 461.00-461.09, 462.02, 463.01, 481,99-482.98, 501.99, 508.00-508.02, 510.01-510.09, 511.10, 513.99, 460.99, 464.01-464.09, 465.99, 470.99-473.99, 480,99, 508.03, 462.01, 462.09, 463.09, 466.99, 483.99-486.09, 502.00-503.09, 519.92, 490.99-491.09
	ICD-9	010-012W, 031A, 033-034B, 052A, 055B, 112E, 122B, 460-466, 475, 481- 482X, 510-X, 511B, 513-B, 480-X, 487-W, 462, 463, 466-B, 473-X, 483, 485, 486, 490, 491B
	ICD-10	A15-16, A20.2, A21.2, A22.1, A31.0, A37, A38, A48.1, B00.2, B01.2, B05.2, B27, B37.1, B39-42, B44, B45.0, B46.0, B58.3, B59, J01-01.9, J02.0, J03.0, J13-15.9, J16.0, J20.0-20.2, J34.0, J36, J39.0-39.1, J85.1-85.3, J86- 86.9, J00, J04-06.9, J10-11.8, J12-12.9, J20.3-20.7, J21.0, , J02, J02.8-02.9, J03, J03.8-03.9, J16, J16.8, J18-18.9, J20, J20.8-21, J21.8-21.9, J22, J32- 32.9, J35.0, J37-37.1, J40-42
Skin	ICD-8	017.01-017.09, 110-111, 050-057, 680.00-680.90, 681.00-682.99, 684.00- 684.09, 686.00-686.9
	ICD-9	017A, 031B, 050-057, 074D, 091D, 110-111, 112D, 681-682X, 683, 684, 685-686X, 680A
	ICD-10	A18.4, A20.0, A22.0, A26.0, A31.1, A32, A36.3, B00-09, B35-36, B37.2, B43.0, B43.2, B45.2, B46.3, B55.1, L00, L01-01.1, L02-02.9, L03-03.9, L04-08.9, L70.0, L30.3
Genitourinary	ICD-8	090-099, 016, 054.02, 590.00-590.99, 595.00-595.02, 597.00, 599.02, 601.00, 604.00, 604.01, 607.30, 611.00, 611.01, 612.01-614.99, 616.00- 616.03, 620.00-620.99, 622.00-622.19, 629.40
	ICD-9	016, 054B, 112B, C, 090-099, 131A, 590-X, 597A, 595-D, X, 597W, 599A, 601-D, 603B, 604A, 604X, 607B, C, 608A, E, 611A, 614-F, W-X, 615A, X, 616-X
	ICD-10	A18.0-18.1, A50-64, A70-74, B37.3-37.4, N10-12, N13.6, N15.1, N15.9, N30-30.3, N30.8-30.9, N34-34.1, N39.0, N41-41.3, N43.1, N45.0-45.9, N48.1-48.2, N49-49.9, N61, N70-76.8, N98.0
Gastrointestinal	ICD-8	000-009, 014, 039.92, 127.99, 522.50, 527.30, 528.00, 528.30, 562.00- 562.19, 566.00-566.01, 567.00-567.02, 569.00, 577.01, 540.00-540.99, 572,99
	ICD-9	001-009, 123, 123, 127, 129, 014, 522E, H, 526E, 527D, 528A, D, 540B, 562-B, 566, 567-C, 569F, 575A, 540A, X, 572A
	ICD-10	001-009, 123, 123, 127, 129, 014, 522E, H, 526E, 527D, 528A, D, 540B, 562-B, 566, 567-C, 569F, 575A, 540A, X, 572A

[a] Reproduced from Khandaker et al.[7]
[b] In Sweden, ICD-8 was used from 1969 to 1986, ICD-9 from 1987 to 1996, and ICD-10 from 1997 onwards.
All diagnoses of post-infection or sequel are excluded.
*The additional codes, B95-97.8, denotes the infecting organism. Bacteria= B95-96.8, Virus= B97- 97.8

© 2020 Lewis G et al. *JAMA Psychiatry.*

eTable 10. Univariable and Multivariable Change in IQ Score According to Population Density and Deprivation, Excluding Those Diagnosed With Nonaffective Psychosis Within 2 years of Conscription (n = 142)

Exposure variable	IQ score (n=227,287)								
	Unadjusted[c]			Bivariable[d]			Fully-adjusted[e]		
	Change in IQ score	95% CI	p	Change in IQ score	95% CI	p	Change in IQ score	95% CI	p
**Population density[a]**	.15	.03 to .16	.014	.61	.50 to .72	<.0001	.06	-.03 to .14	.191
**Deprivation[b]**	-1.58	-1.68 to -1.48	<.0001	-1.72	-1.82 to -1.62	<.0001	-.70	-.78 to -.62	<.0001

Path A of mediation model, eFigure 1
[a] Units are number of people per km^2 and unit change is per one standard deviation
[b] Units are based on the deprivation index and unit change is per one standard deviation
[c] Separate univariable models for population density and deprivation
[d] Bivariable model for population density and deprivation
[e] Population density and deprivation adjusted for paternal age at birth, family income, maternal education, paternal education, any psychosis or bipolar in parents, parent/s born outside of Sweden.

© 2020 Lewis G et al. *JAMA Psychiatry.*

eTable 11. Univariable and Multivariable Odds Ratios (OR) for Associations Between Population Density, Deprivation, IQ, and Nonaffective Psychosis, Multiply Imputed Sample and Further Adjusted for Paternal IQ

Exposure variable	Non-affective psychosis (n=282,232)											
	Unadjusted[d]			Bivariable[e]			Multivariable[f]			Fully-adjusted[g]		
	OR	95% CI	p	OR	95% CI	p	OR	95% CI	p	OR	95% CI	p
**Population density[a]**	1.17	1.13 to 1.21	<.0001	1.13	1.09 to 1.17	<.0001	1.09	1.05 to 1.13	<.0001	1.09	1.05 to 1.13	<.0001
**Deprivation[b]**	1.18	1.14 to 1.23	<.0001	1.14	1.09 to 1.18	<.0001	1.08	1.04 to 1.13	.001	1.06	1.02 to 1.10	.008
**IQ[c]**	0.73	0.69 to 0.76	<.0001	-	-	-	-	-	-	0.71	0.68 to 0.75	<.0001

[a] Units are number of people per km^2 and unit change is per one standard deviation (3863.97 people per km^2)
[b] Units are based on the deprivation index and unit change is per one standard deviation (1.96 points)
[c] Units are based on the Wechsler Adult Intelligence Scale unit change is per one standard deviation (15 points)
[d] Separate univariable models for population density, deprivation and IQ
[e] Bivariable model for population density and deprivation
[f] Population density and deprivation adjusted for paternal age at birth, family income, maternal education, paternal education, any psychosis or bipolar in parents, parent/s born outside of Sweden and paternal IQ.
[g] The above multivariable model, also with IQ

© 2020 Lewis G et al. *JAMA Psychiatry.*

eTable 12. Power Simulations for Interaction Between Population Density and Deprivation at Birth and IQ on Risk of Nonaffective Psychosis for Various Hypothesized Effect Sizes

	Interaction odds ratio[1]		
	**1.01**	**1.05**	**1.10**
**Interaction term[a]**	**Power (%)**	**Power (%)**	**Power (%)**
Population density by IQ	19.4	98.4	100.0
Deprivation by IQ	14.4	99.4	100.0

[a]Interaction odds ratios refer to hypothetical interaction odds ratios for the interaction term between either population density and IQ or deprivation and IQ, respectively. In observed data (N=227,429) the interaction odds ratio for these terms was 1.01 (95%CI: 0.97 to 1.06) for both population density by IQ and deprivation by IQ. We ran power simulations based on these observed interaction effect sizes as well as hypothetical interaction odds ratios of 1.05 and 1.10. Power simulations were based on models mutually adjusted for the other interaction. We set correlations between each variable as: population density & deprivation = 0.28; population density & IQ = 0.01; deprivation & IQ = -0.12.

© 2020 Lewis G et al. *JAMA Psychiatry.*

eReferences

1. David AS, Malmberg A, Brandt L, Allebeck P, Lewis G. IQ and risk for schizophrenia: A population-based cohort study. *Psychol Med*. 1997;27(6):1311-1323. doi:10.1017/S0033291797005680
2. Sipos A, Rasmussen F, Harrison G, et al. Paternal age and schizophrenia: a population based cohort study. *BMJ*. 2004;329(7474):1070. doi:10.1136/bmj.38243.672396.55
3. Ludvigsson JF, Andersson E, Ekbom A, et al. External review and validation of the Swedish national inpatient register. *BMC Public Health*. 2011;11:450. doi:10.1186/1471-2458-11-450
4. Price C, Dalman C, Zammit S, Kirkbride JB. Association of Residential Mobility over the Life Course with Nonaffective Psychosis in 1.4 Million Young People in Sweden. *JAMA Psychiatry*. 2018;75(11):1128-1136. doi:10.1001/jamapsychiatry.2018.2233
5. Cannon M, Jones PB, Murray RM. Obstetric complications and schizophrenia: historical and meta-analytic review. *Am J Psychiatry*. 2002;159(7):1080-1092.
6. Seidman LJ, Buka SL, Goldstein JM, Horton NJ, Rieder RO, Tsuang MT. The relationship of prenatal and perinatal complications to cognitive functioning at age 7 in the New England Cohorts of the National Collaborative Perinatal Project. *Schizophr Bull*. 2000;26(2):309-321. doi:10.1093/oxfordjournals.schbul.a033455
7. Khandaker GM, Dalman C, Kappelmann N, et al. Association of Childhood Infection With IQ and Adult Nonaffective Psychosis in Swedish Men. *JAMA Psychiatry*. 2018;75(4):356-362. doi:10.1001/jamapsychiatry.2017.4491
8. MacCabe JH, Lambe MP, Cnattingius S, et al. Scholastic achievement at age 16 and risk of schizophrenia and other psychoses: a national cohort study. *Psychol Med*. 2008;38(8):1133-1140. doi:S0033291707002048 [pii] 10.1017/S0033291707002048
9. Hernán MA. A definition of causal effect for epidemiological research. *J Epidemiol Community Health*. 2004;58(4):265-271. doi:10.1136/JECH.2002.006361
10. Maldonado G, Greenland S. Estimating causal effects. *Int J Epidemiol*. 2002;31(2):422-429. http://www.ncbi.nlm.nih.gov/pubmed/11980807. Accessed October 22, 2018.
11. Imai K, Keele L, Tingley D. A general approach to causal mediation analysis. *Psychol Methods*. 2010;15(4):309-334. doi:10.1037/a0020761
12. Luedicke J. powersim: simulation-based power analysis for linear and generalized linear models. August 2013.

© 2020 Lewis G et al. *JAMA Psychiatry.*
